# Consensus proposals for classification of the family *Hepeviridae*

**DOI:** 10.1099/vir.0.068429-0

**Published:** 2014-10

**Authors:** Donald B. Smith, Peter Simmonds, members of the International Committee on the Taxonomy of Viruses Hepeviridae Study Group, Shahid Jameel, Suzanne U. Emerson, Tim J. Harrison, Xiang-Jin Meng, Hiroaki Okamoto, Wim H. M. Van der Poel, Michael A. Purdy

**Affiliations:** 1 University of Edinburgh, Centre for Immunity, Infection and Evolution, Edinburgh, Scotland, UK; 2 Wellcome Trust/DBT India Alliance, Hyderabad, India; 3 Special Volunteer, Retired Head Molecular Hepatitis Section, National Institute of Allergy and Infectious Diseases, National Institutes of Health, Bethesda, MD, USA; 4 University College of London, London, UK; 5 College of Veterinary Medicine, Virginia Polytechnic Institute and State University, Blacksburg, VA, USA; 6 Division of Virology, Department of Infection and Immunity, Jichi Medical University School of Medicine, Tochigi-ken, Japan; 7 Central Veterinary Institute, Wageningen University and Research Centre, Lelystad, The Netherlands; 8 Centers for Disease Control and Prevention, National Center for HIV/Hepatitis/STD/TB Prevention, Division of Viral Hepatitis, Atlanta, GA, USA

## Abstract

The family *Hepeviridae* consists of positive-stranded RNA viruses that infect a wide range of mammalian species, as well as chickens and trout. A subset of these viruses infects humans and can cause a self-limiting acute hepatitis that may become chronic in immunosuppressed individuals. Current published descriptions of the taxonomical divisions within the family *Hepeviridae* are contradictory in relation to the assignment of species and genotypes. Through analysis of existing sequence information, we propose a taxonomic scheme in which the family is divided into the genera *Orthohepevirus* (all mammalian and avian hepatitis E virus (HEV) isolates) and *Piscihepevirus* (cutthroat trout virus). Species within the genus *Orthohepevirus* are designated *Orthohepevirus* A (isolates from human, pig, wild boar, deer, mongoose, rabbit and camel), *Orthohepevirus* B (isolates from chicken), *Orthohepevirus* C (isolates from rat, greater bandicoot, Asian musk shrew, ferret and mink) and *Orthohepevirus* D (isolates from bat). Proposals are also made for the designation of genotypes within the human and rat HEVs. This hierarchical system is congruent with hepevirus phylogeny, and the three classification levels (genus, species and genotype) are consistent with, and reflect discontinuities in the ranges of pairwise distances between amino acid sequences. Adoption of this system would include the avoidance of host names in taxonomic identifiers and provide a logical framework for the assignment of novel variants.

## Introduction

Hepatitis E virus (HEV) is the cause of a self-limiting hepatitis with mortality rates of <2 % for immune competent individuals. However, higher mortality rates (10–30 %) are observed amongst pregnant women, while infection may become chronic in immunocompromised individuals. HEV was first recognized in the 1980s and its nucleotide sequence published in the 1990s (Huang *et al.*, 1992; Reyes *et al.*, 1990; Tam *et al.*, 1991; Tsarev *et al.*, 1992). Since then, HEV variants have been detected in a variety of human populations and in potential zoonotic sources such as pig, wild boar, deer, rabbit and mongoose. The variants that infect humans have been classified into four genotypes. Genotypes 1 and 2 are transmitted faecal–orally between humans, and genotypes 3 and 4 may be transmitted to humans zoonotically from infected pigs, deer and wild boar. These genotypes have been subdivided further into numerous subtypes (Lu *et al.*, 2006), although the underlying criteria are controversial (Okamoto, 2007; Smith *et al.*, 2013). A more distantly related virus detected in chickens also has been divided into genotypes (Bilic *et al.*, 2009; Hsu & Tsai, 2014; Huang *et al.*, 2004).

This relatively simple taxonomical landscape was disturbed recently by the discovery of viruses that are related to human HEV but infect rabbits (Zhao *et al.*, 2009) and wild boar (Takahashi *et al.*, 2011). Also, more divergent, HEV-like viruses have been described in rats (Johne *et al.*, 2010a), ferrets (Raj *et al.*, 2012) and bats (Drexler *et al.*, 2012), and an even more divergent virus has been isolated from cutthroat trout (Batts *et al.*, 2011). This last virus has a genome organization similar to that of HEVs, but shares very low levels of nucleotide and amino acid sequence identity. Finally, a virus detected in sewage has a partial genome sequence suggesting that it may represent a further, highly divergent member of the family *Hepeviridae* (Ng *et al.*, 2012).

Several recent papers have attempted to summarize this diversity, but have unfortunately reached different conclusions, resulting in the use of multiple, contradictory definitions in the literature. For example, some authors have argued that avian HEV, bat HEV and cutthroat trout virus should be considered as belonging to different genera (Meng, 2013), while others have suggested that avian HEV, bat HEV and rat HEV should be considered as species within a single genus, with cutthroat trout virus as the sole member of a distinct genus (Smith *et al.*, 2013). Both schemes are out-of-date in light of the recent publication of sequences from divergent variants isolated from moose (Lin *et al.*, 2014), mink (Krog *et al.*, 2013), fox (Bodewes *et al.*, 2013), ferret (Li *et al.*, 2014), wild boar (Takahashi *et al.*, 2014) and camel (Woo *et al.*, 2014).

The purpose of this paper is to present a consensus taxonomic framework that provides an agreed basis for the classification of currently described HEV variants, taking into account phylogenetic relationships, the extent of sequence identity and host range. This framework will facilitate the future classification of novel members of this family by providing researchers with straightforward guidelines for assigning new HEV variants. It also helps to clarify the zoonotic threat of particular HEV variants, so that, for example, rat HEV, which is a variant that has not so far been detected in humans, is clearly distinguished from variants of human HEV genotype 3 that are also found in rats (Lack *et al.*, 2012). We propose the use of a common reference sequence and numbering system to simplify comparisons between different studies.

Our model for this consensus approach is that of *Hepatitus C virus* (HCV), in which the adoption of a consensus classification system (Simmonds *et al.*, 2005) and numbering system with respect to a reference sequence (Kuiken *et al.*, 2006) have stabilized the terminology used in HCV research and assisted researchers in providing unique and rational attribution of genotypes and subtypes. The utility of this framework is illustrated by the fact that an update to the HCV classification system almost 10 years later (Smith *et al.*, 2014) was required simply to accommodate the large number of subsequently assigned subtypes, whereas only minor changes to the consensus rules for genotype and subtype assignment were necessary.

## Results and Discussion

### Genera

Members of the family *Hepeviridae* are positive-stranded RNA viruses with genomes of 6.6 to 7.3 kb. The longest ORF (ORF1) encodes a non-structural protein with several distinct domains: methyltransferase, Y-domain, papain-like protease, polyproline region (also known as the hypervariable region; HVR), macro domain, helicase and RNA-dependent RNA polymerase. ORF1 is followed by ORF2, which encodes the capsid protein, and ORF3, which overlaps with ORF2 and encodes a phosphoprotein that modulates cellular activities.

**Fig. 1.**
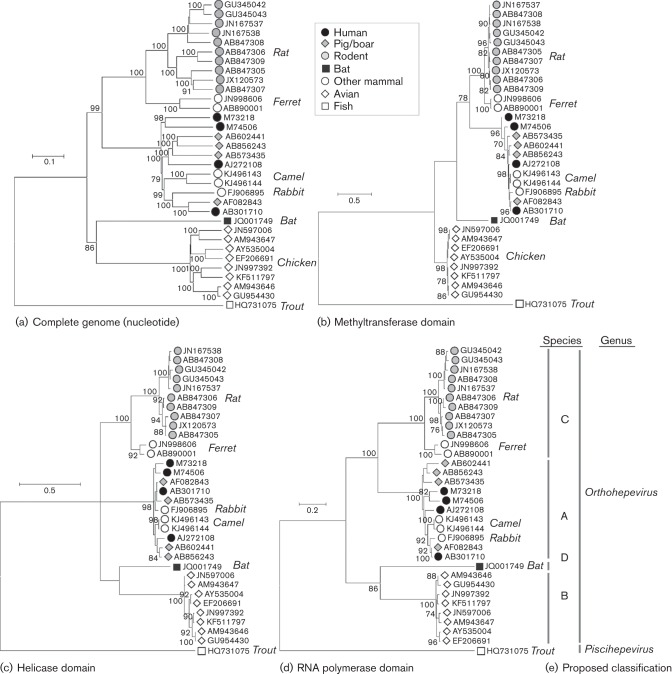
Phylogenetic analysis of members of the family *Hepeviridae*. (a) Neighbour-joining tree of p-distances among aligned complete genome sequences. (b) Maximum-likelihood tree for amino acid sequences in methyltransferase domain (ORF1-28 to ORF1-389) (c) helicase domain (ORF1-971 to ORF1-1185) (d) RNA-dependent RNA polymerase domain (ORF1-1249 to ORF-1-1671). (e) Proposed classification. Maximum-likelihood trees were computed using the model according to Le & Gascuel with a gamma distribution of evolutionary rates among sites with some invariant sites. Branches supported by >70 % of bootstrap replicates are indicated.

Despite this conserved genome structure, phylogenetic analysis within the family is complicated by difficulty in aligning the genome sequences of the most divergent variants. In particular, amino acid sequence identities between cutthroat trout virus and other members of the family are only 26–27 % (ORF1), 18–21 % (ORF2) and 13–16 % (ORF3). In contrast, identities between avian, rat and human HEV stand at 42–49 %, 42–55 % and 20–29 %, respectively (Batts *et al.*, 2011). These average figures mask the fact that, in some genomic regions, no credible amino acid sequence alignment is achievable (Drexler *et al.*, 2012; Huang *et al.*, 2004; Johne *et al.*, 2010b; Smith *et al.*, 2013). Hence, phylogenetic comparisons simply based on pairwise (p)-distances between complete genome nucleotide sequences (Fig. 1a) may give a distorted impression of the relationships between variants. In addition, phylogenetic analysis must take into account the fact that substitutions at synonymous sites are saturated even in comparisons between the different genotypes of human HEV (Smith *et al.*, 2013).

**Fig. 2.**
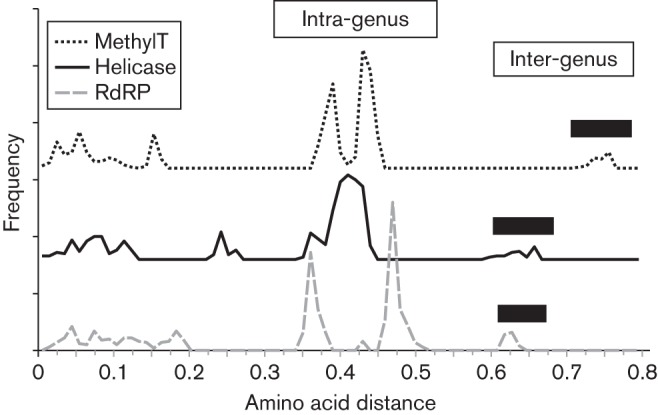
Frequency distribution of distances among the sequences of members of the family *Hepeviridae*. Plots show the frequency of amino acid sequence p-distances among amino acid sequences of the methyltransferase (ORF1-28 to ORF1-389, dotted line), helicase (ORF1-971 to ORF1-1185, solid line) and RdRP (ORF1-1249 to ORF1-1671, dashed line) domains. The methyltransferase and helicase distance frequencies are shifted by 160 and 80 respectively in order to improve legibility. Filled bars indicate the range of inter-generic distances.

To recover more accurately sequence relationships between the most divergent variants in the *Hepeviridae*, we screened for regions of the genome that are clearly homologous using the Motif Scan program (http://myhits.isb-sib.ch). This identified three subgenomic regions in ORF1 comprising the methyltransferase [ORF1 residues 28 (ORF1-28) to ORF1-389 numbered relative to the sequence of the HEV Burma isolate, GenBank accession M73218], helicase (ORF1-971 to ORF1-1185) and RNA-dependent RNA polymerase (ORF1-1249 to ORF1-1671). Maximum-likelihood analysis of alignments of each region reproduced phylogenetic trees with a similar topology but with much shorter terminal branches (Fig. 1b–d) than obtained from complete genome sequences. Branch lengths in these trees likely represent better reconstructions of evolutionary depth and demonstrate that cutthroat trout virus is substantially more divergent from other members of the family *Hepeviridae* than indicated by nucleotide sequence comparisons. Similarly, p-distances amongst these subgenomic amino acid sequences form non-overlapping distributions, with the greatest distances observed in comparisons including cutthroat trout virus (Fig. 2). In addition, cutthroat trout virus has an aberrant genome organization (ORF3 is displaced towards the middle of ORF2) and a distinct host range (fish rather than mammals or birds). For these reasons, we recommend that cutthroat trout virus should be assigned to the new genus *Piscihepevirus* (Table 1), as proposed previously (Meng, 2013), with the single species *Piscihepevirus A*. We prefer these genus and species names to *Cutrovirus* (Batts *et al.*, 2011), since the virus appears to be confined to fish, having also been detected in five other species of trout (Batts *et al.*, 2011).

**Table 1.** Proposed classification of the family *Hepeviridae*

Family	Genus	Species	Prototype isolate	GenBank accession	Predominant host species	Genotype	Reference strain	Reference accession
*Hepeviridae*			Burma	M73218	Human			
	*Orthohepevirus*		Burma	M73218	Human			
		*Orthohepevirus* A	Burma	M73218	Human	HEV-1	Burma	M73218
					Human	HEV-2	Mexico	M74506
					Human, pig, rabbit, deer, mongoose	HEV-3	Meng	M74506
					Human, pig	HEV-4	T1	AJ272108
					Wild boar	HEV-5	JBOAR135-Shiz09	AB573435
					Wild boar	HEV-6	wbJOY_06	AB602441
					Camel	HEV-7	DcHEV-178C	KJ496143
		*Orthohepevirus* B	F93-5077	AY535004	Chicken			
		*Orthohepevirus* C	R63	GU345042	Rat	HEV-C1	R63	GU345042
					Ferret	HEV-C2	FRHEV4	JN998606
		*Orthohepevirus* D	BatHEV/BS7/GE/2009	JQ001749	Bat			
	*Piscihepevirus*		Heenan Lake	HQ731075	Trout			
		*Piscihepevirus* A	Heenan Lake	HQ731075	Trout			

We propose that the remaining HEV variants should be considered as members of the genus *Orthohepevirus* (Table 1), extending the usage of a previous proposal (Meng, 2013). This change of genus name from *Hepevirus* is preferred because it makes it clear that not all members of the *Hepeviridae* belong to the same genus.

Analysis of part of the RdRP domain from an incomplete virus genome sequence (Hepelivirus) obtained from raw sewage suggests that this may represent an additional genus within the family *Hepeviridae* (Ng *et al.*, 2012). However, a complete genome sequence would be required to confirm the taxonomic position of this virus, for which the host range remains unknown.

### Species

**Fig. 3.**
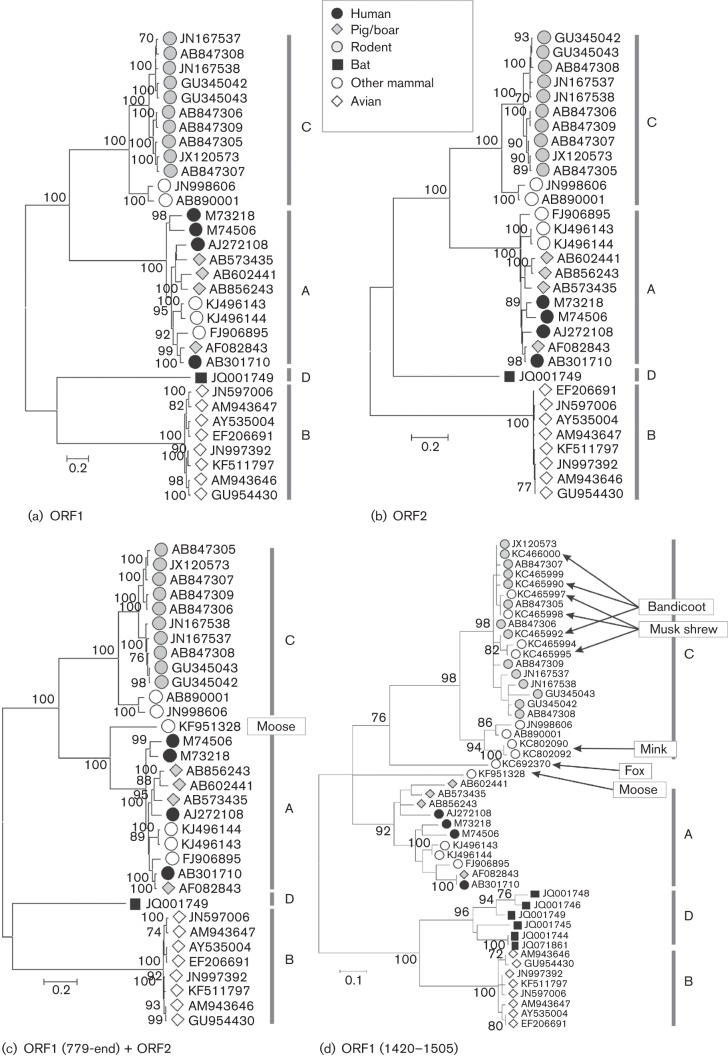
Phylogenetic analyses of members of the genus *Orthohepevirus*. Maximum-likelihood tree for amino acid sequences in (a) ORF1 and (b) ORF2, (c) ORF1-779 to the end of ORF2 with the addition of KF951328 from moose, (d) ORF1-1420 to ORF1-1505 with the addition of sequences from mink, fox, greater bandicoot, Asian musk shrew and bat. Maximum-likelihood trees were computed using the model according to Le & Gascuel with a gamma distribution of evolutionary rates among sites, with some invariant sites. Branches supported by >70 % of bootstrap replicates are indicated.

The next obvious level of sequence diversity amongst members of the genus *Orthohepevirus* is that which separates variants originally isolated from different host species (Fig. 3). For example, all isolates from chickens belong to one clade, as do those from rats and ferrets, those from humans, deer, mongooses, pigs, wild boar and camels, while a single complete genome sequence isolated from a bat forms a fourth group. These four groupings are observed regardless, whether the analysis is performed on complete genome nucleotide sequences or subgenomic amino acid sequences (Figs 1 and 3). We propose that these groupings correspond to four species and that they be designated *Orthohepevirus* A to *Orthohepevirus* D (Table 1).

Subgenomic sequences of variants isolated from other mammalian species have recently become available but phylogenetic analyses are complicated because they overlap with only relatively short regions. The most complete sequence (5081 nt) is derived from a variant isolated from a moose (Lin *et al.*, 2014). Phylogenetic analysis of the amino acid sequence of this isolate for part of ORF1 and all of ORF2 (Fig. 3) shows that it is distinct from all other variants, although it shares a branch with species *Orthohepevirus* A in the same way that the ferret variants group with rat-derived species *Orthohepevirus* C. Similar analysis of virus sequences isolated from mink (Krog *et al.*, 2013) shows that these group with sequences isolated from ferrets. A sequence isolated from a fox (Bodewes *et al.*, 2013) appears to be more distinct (Fig. 3d) and could represent an additional species. However, this last analysis was based on a relatively short subgenomic region (ORF1-1420 to ORF1-1505) in which the grouping of species *Orthohepevirus* C isolates from rat and ferret, and of the moose variant with *Orthohepeviru*s A variants from human, pig and wild boar, were less distinct than for comparisons based on longer coding regions (Fig. 3). We recommend that assignment of the moose and fox variants to particular *Orthohepevirus* species should not be made until comparisons based upon complete genome sequences are available.

An advantage of using species names of the form *Orthohepevirus* A*,* etc., rather than names based on avian HEV, rat HEV or bat HEV, is that they will not be compromised by current or future discoveries on the extent of host range. For example, variants of species *Orthohepevirus* C have been isolated from *Rattus* (Li *et al.*, 2013) and *Bandicota* species (the greater bandicoot rat) (Li *et al.*, 2013), which are members of the order Rodentia, but also from Asian musk shrews (Guan *et al.*, 2013), which are members of the order Soricomorpha, and from ferrets (Li *et al.*, 2014; Raj *et al.*, 2012) and mink (Krog *et al.*, 2013), which are members of the order Carnivora. Wider screening for members of this species, and also for members of species *Orthohepevirus* B and *Orthohepevirus* D, may reveal wider host ranges than those recognized at present.

### Genotypes

Although the International Committee on Taxonomy of Viruses does not provide official designations for taxonomic entities below the species level, it is useful for researchers to have an agreed designation of genotypes within each species grouping.

#### *Orthohepevirus* A.

Within species *Orthohepevirus* A, four genotypes are currently described that infect humans (HEV-1, HEV-2, HEV-3 and HEV-4), and assignment of complete genome sequences to these genotypes is generally unambiguous. The only exceptions are recombinant viruses, which have been documented both within and between genotypes (Chen *et al.*, 2012; van Cuyck *et al.*, 2005; Fan, 2009; Wang *et al.*, 2010), although in some cases recombinant viruses may represent laboratory artefacts (Wang *et al.*, 2010). More problematic is the designation of additional genotypes HEV-5 and HEV-6, names which have been variously assigned to avian HEV and rat HEV (here proposed to be classified as members of the species *Orthohepevirus* B and *Orthohepevirus* C, respectively) and variants isolated from wild boar (Oliveira-Filho *et al.*, 2013; Smith *et al.*, 2013; Takahashi *et al.*, 2011), or by implication to variants isolated from rabbits (Geng *et al.*, 2011; Zhao *et al.*, 2009).

**Fig. 4.**
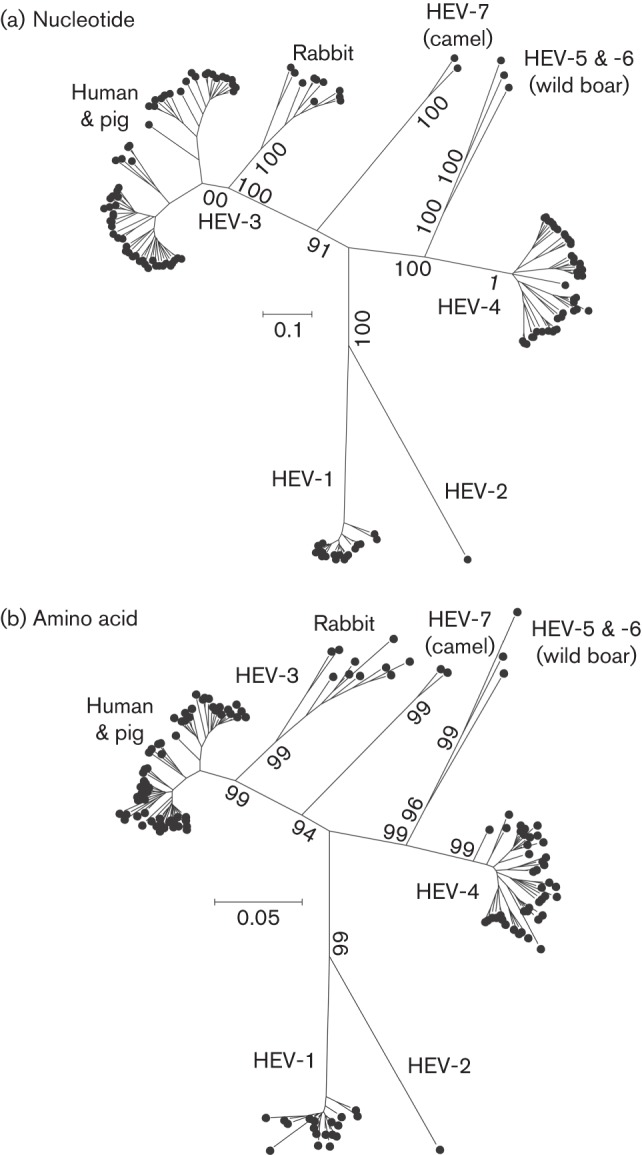
Phylogenetic analyses of members of *Orthohepevirus* A. Maximum-likelihood trees were produced for 137 concatenated ORF1 and ORF2 regions (excluding the HVR and rabbit HEV-specific insertion) for (a) nucleotide sequences using the general time-reversible model with gamma distribution and invariant sites and (b) amino acid sequences using the Jones–Taylor–Thornton model with frequencies, and gamma distribution with invariant sites. Branches supported by >70 % of bootstrap replicates are indicated.

Phylogenetic analysis of the nucleotide and amino acid sequences of concatenated ORF1 and ORF2 (excluding the HVR and the hypervariable insertion found in all rabbit HEV isolates) for these variants, including a divergent variant recently described from wild boar (Takahashi *et al.*, 2014), reveals four fully supported branches consisting of (i) HEV-1 and HEV-2, (ii) HEV-3 and variants isolated from rabbits, together with a closely related patient sequence, and (iii) HEV-4 together with all three isolates from wild boar and (iv) isolates from camels (Fig. 4). However, given the precedence of HEV-1 and HEV-2 forming well recognized and phylogenetically distinct genotypes, the least disruptive way of representing these phylogenetic relationships would be to retain the HEV-1, HEV-2, HEV-3 and HEV-4 genotype assignments.

**Fig. 5.**
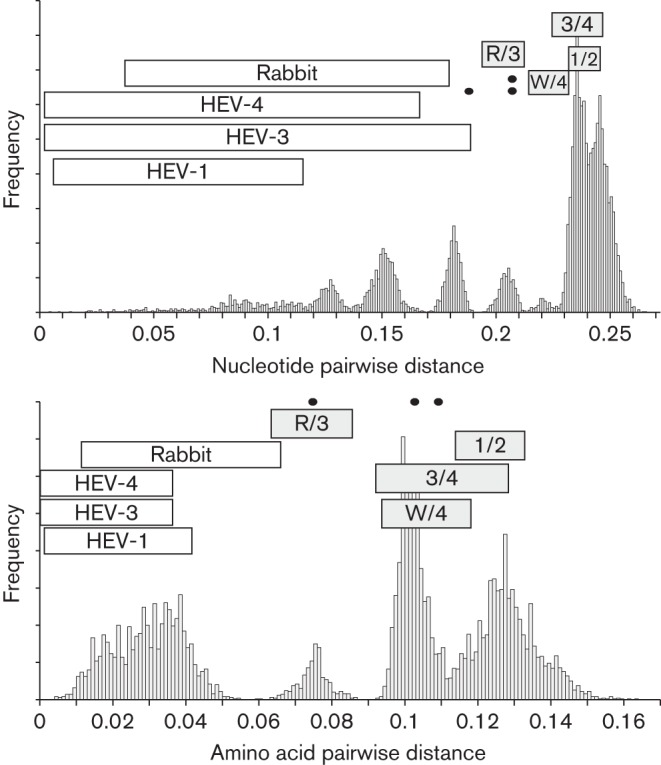
Frequency distribution of distances among sequences of variants in the genus *Orthohepevirus*. Histograms show the frequency of nucleotide (a) and amino acid sequence p-distances (b) among 135 concatenated ORF1 and ORF2 sequences (excluding the HVR and rabbit HEV-specific HVR insertion). Maximum and minimum intra-genotype ranges of distances within genotypes 1, 3 and 4 and between rabbit sequences are indicated with open bars. Grey-filled bars depict distances between HEV-3 and rabbit variants (R/3) and inter-genotype distances (3/4, HEV-3 and HEV-4; 1/2, HEV-1 and HEV-2; W/4, wild boar and HEV-4). Distances between the three wild boar sequences are indicated with spots.

Variants derived from rabbits (and the single related human isolate JQ013793) show a wider range of amino acid sequence distances from each other (maximum value 0.081) than HEV-1, -3 and -4 (maximum values 0.041, 0.053 and 0.053 respectively) while minimum distances between them and HEV-3 variants are lower (0.061) than between rabbit sequences and other HEV genotypes or variants (minimum value 0.108). A previous study discussed whether rabbit viruses might be assigned to genotype 3 as divergent members or form a separate genotype, since nucleotide and amino acid distances were intermediate between those observed within HEV-1, HEV-3 and HEV-4 and distances between genotypes (Smith *et al.*, 2013). Our analysis here of further rabbit-derived HEV variants (JX121233, JQ768461, JX109834, AB740220, AB740221, AB740222 and JX565469) confirms this finding, for example, the pairwise amino acid distances with genotype 3 that overlap those between genotype 3 and 4. However, the most extreme distances between rabbit HEV and HEV-3 involve the isolates FJ906895 and FJ906896. These sequences contain numerous amino acid substitutions clustered at the C-terminus (FJ906895) or N-terminus (FJ906896), which are at sites that are otherwise highly conserved throughout *Orthohepevirus* A sequences, and so are likely to represent sequencing artefacts. Excluding these two sequences from the analysis of pairwise distances, there was no overlap between amino acid distances between rabbit and HEV-3 sequences, and between HEV-3 and HEV-4 (Fig. 5). Retaining these isolates but excluding the terminal regions containing the aberrant sites from the *Orthohepevirus* alignment similarly eliminates overlap between these categories (data not shown). Since HEV-3 and the rabbit HEV also share a long branch on phylogenetic analysis, we consider it simplest to provisionally assign the rabbit sequences to genotype HEV-3. 

On this basis, amino acid distances of concatenated ORF1 and ORF2 (lacking hypervariable regions) greater than 0.088 could then act as a threshold to demarcate intra- and inter- genotype distances. Using this criterion, the three wild boar isolates would comprise two additional genotypes HEV-5 (AB573435) and HEV-6 (AB602441 and AB856243 differing from each other by 0.076 and from HEV-5 by >0.10), while the variants isolated from camels (differing from all other sequences by >0.095) would become HEV-7.

The virus isolated from moose appears to constitute a distinct lineage within species *Orthohepevirus* A, but definitive placement requires a complete genome sequence.

#### *Orthohepevirus* B.

Although four genotypes of *Orthohepevirus* B have been proposed (Bilic *et al.*, 2009; Hsu & Tsai, 2014; Huang *et al.*, 2004), these are much less diverse than the genotypes of *Orthohepesvirus* A. For example there is <6 % divergence in *Orthohepevirus* B complete genome amino acid sequences (Figs 1 and 3), which is less than the divergence observed within HEV-3 (<9 %) and HEV-4 (<7 %). In addition, the amino acid sequence distances among the eight currently available complete genome sequences of members of species *Orthohepevirus* B form a continuous distribution, where distances within genotypes (maximum 3.3 %) approach those between genotypes (minimum 3.6 %). If additional complete genome sequences were available, this narrow division might disappear.

#### *Orthohepevirus* C.

The extent of diversity (<11 %) among complete genome amino acid sequences of the rat-derived *Orthohepevirus* C variants barely overlaps that observed among genotypes of species *Orthohepevirus* A (10–18 %). However, much greater divergence is observed between rat and ferret *Orthohepevirus* C variants (23 %). Analysis of the short region of ORF1 for which sequence information is available from additional variants (Fig. 2d) indicates that diversity among greater bandicoot and Asian musk shrew isolates falls within that of the rat variants (Guan *et al.*, 2013; Li *et al.*, 2013), while isolates from the mink group falls within those from ferret (Krog *et al.*, 2013). Based on this information, we propose that *Orthohepevirus* C may be divided into two genotypes, namely HEV-C1, including isolates derived from hosts in the orders Rodentia and Soricomorpha, and HEV-C2, including isolates derived from ferret (and possibly mink).

#### *Orthohepevirus* D.

A single complete genome sequence is available for species *Orthohepevirus* D, but phylogenetic analysis of a short region of ORF2, for which data from additional isolates are available, suggests a level of diversity equivalent to that within species *Orthohepevirus* A and *Orthohepevirus* C (Fig. 2d). While this is consistent with the existence of multiple genotypes within *Orthohepevirus* D, additional sequence information is required to confirm that these relationships prevail for larger genomic regions.

### Subgenotypes

Several studies have attempted to define subgenotypes of genotypes HEV-1, HEV-3 and HEV-4 in the species *Orthohepevirus* A, in some instances based on the analysis of subgenomic regions (Lu *et al.*, 2006; Zhu *et al.*, 2011). Such categories may form useful labels for epidemiological studies (Dai *et al.*, 2013), but more recent analysis of complete genome sequences suggests that it is not possible to define discrete boundaries that distinguish subgenotypes with consistency (Okamoto, 2007; Oliveira-Filho *et al.*, 2013; Smith *et al.*, 2013). We recommend the approach, commonly adopted in several recent publications, of labelling clades apparent within sequence sets (Dai *et al.*, 2013; Ijaz *et al.*, 2014; Oliveira-Filho *et al.*, 2013) without defining them as permanent classification assignments.

### Reference sequences and numbering

A recurring difficulty in the literature, relating to molecular studies of members of the family *Hepeviridae,* is that of comparing different studies for which there is no explicit standard sequence with reference to which nucleotides or amino acid residues are numbered. The presence of numerous insertions or deletions and regions of low similarity in alignments of *Hepeviridae* sequences precludes a unified numbering system that is applicable across all species or genera. We recommend that genome sequences be numbered with reference to the first nucleotide of the prototype complete genome sequence available for each species within the genus *Orthohepevirus* (Table 1). Nucleotide sites in variants that contain insertions relative to the prototype sequence should be identified with additional letters, beginning at the site of insertion. For example, a three-nucleotide insertion at position 1788 of the prototype sequence would be numbered 1788a, 1788b, 1788c. Insertions of more than 26 nt would be numbered from the twenty-seventh position as 1788aa, 1788ab, etc. and then as 1788ba, 1788bb, etc. as required. This mirrors the system adopted for HCV (Kuiken *et al.*, 2006). Similarly, amino acid residues should be numbered with reference to the first residue of the appropriate ORF from the reference sequence, for example ORF1-929, with an insertion at this site indicated by suffix letters such as ORF1-929a, ORF1-929b, etc., followed if necessary by ORF1-929 aa, ORF1-929ab, etc.

### Conclusions

The proposed classification, which assigns a separate hierarchy of genus and species, respects the different levels of divergence between the cutthroat trout virus and all other hepeviruses. The degrees of sequence divergence and conservation of genomic features associated with these categories closely match those attributed to genera and species in other virus families (e.g. *Picornaviridae, Caliciviridae* and *Flaviviridae*). This description of relationships among members of the family *Hepeviridae* will help to resolve current confusion in the literature and, by providing a rational basis for taxonomic assignments, help to reduce the number of future conflicts as more members of this family are discovered.

## Methods

Phylogenetic analysis included the complete genome sequences in GenBank accessions M73218, M74506, AF082843, FJ906895, AB301710, AJ272108, AB602441, AB856243, AB573435, KJ496143, KJ496144, JN167537, JN167538, GU345042, GU345043, JX120573, AB847305, AB847306, AB847307, AB847308, AB847309, JN998606, JN998607, AB890001, AB890374, JN597006, JN997392, AM943647, AM943646, GU954430, AY535004, EF206691, KF511797, JQ001749 and HQ731075. Analyses of subgenomic regions included the additional sequences JQ001744-8 and JQ071861 (bat), JN167530-6, KC473527-31, JN040433, KC294199, JF516246, GQ504009-10 and AB725884-900 and AB847310-406 (rat), KF268376-93 (ferret), KC465990-6001 (greater bandicoot and Asian musk rat), KC692369-70 (fox), KC802090-3 (mink), AY043166 (chicken) and KF951328 (moose).

A further dataset included 137 complete genome sequences isolated from humans, pigs, rabbits and wild boar (downloaded from GenBank on 4 February 2014, excluding recombinant sequences and sequences differing from other sequences by less than 0.2 % of nucleotide positions in ORF1), as follows: M73218, AB720034, JF443717, JF443718, JF443720, JF443721, JF443722, JF443723, JF443725, JF443726, JQ655734, FJ457024, D11092, AY204877, AY230202, AF185822, AF076239, X99441, M74506, KC618402, AB780450, AB740232, JQ953664, JQ953665, JQ953666, JN837481, JN906974, AB593690, AB630970, AB630971, AB591733, AB591734, HQ389543, HQ709170, AB481226, AB481228, AB481229, FJ653660, FJ426403, FJ426404, FJ956757, FJ998008, FJ705359, AB291951, AB291953, FJ527832, EU375463, AB189071, AB236320, EU723512, EU723514, AB073912, EU495148, AB089824, AB091394, AB222182, AB222183, AB222184, AB248520, AB248522, AB290312, AB290313, AB291961, AB291962, AB291963, EU723513, EU723516, AB074920, AP003430, EU360977, AB369687, AB369689, AB369691, AB246676, AY575857, AF455784, AY115488, AF060669, KF922359, AF082843, FJ906895, JX109834, AB740220, FJ906896, AB740221, AB740222, JX565469, JQ013791, JQ013792, JQ013793, FJ610232, KC492825, KF176351, JF915746, JQ740781, JX855794, AB291959, JQ655733, JQ655735, JQ655736, AB698654, JQ993308, AB602439, GU361892, GU119960, GU119961, GU206559, HQ634346, AB481227, HM439284, FJ763142, GU188851, AB480825, AB197673, AB197674, AB091395, AB220974, AB291964, AB074915, AB080575, EU676172, AB108537, AB369688, AB369690, EU366959, EF570133, DQ279091, EF077630, AY723745, AB253420, AY594199, AJ272108, AB602441, AB856243, AB573435, KJ496143 and KJ496144. Sequences were aligned using muscle v3.8 (Edgar, 2004) within sse v1.1 (Simmonds, 2012), and then refined manually. Phylogenetic analysis was conducted using mega version 6 (Tamura *et al.*, 2013). Using the optimal model for each dataset, maximum-likelihood trees were reproduced by using the programs Models and Phylogeny in mega 6. Distances between nucleotide and amino acid sequences were generated within sse program. Homology to known protein domains were identified using Motif Scan (http://myhits.isb-sib.ch).
